# On the Implementation of a Compact Vertical DC Biasing Network with Significantly Reduced RF Components for Phase-Shifter-Free Beam Steering

**DOI:** 10.3390/s26113584

**Published:** 2026-06-04

**Authors:** Shuxin Zheng, Bingyi Qian, Xiaoming Chen, Ahmed A. Kishk

**Affiliations:** 1 School of Information and Communications Engineering, Xi’an Jiaotong University, Xi’an 710049, Chinaqianbingyi@stu.xjtu.edu.cn (B.Q.); 2 Department of Electrical and Computer Engineering, Concordia University, Montreal, QC H3G 1M8, Canada

**Keywords:** DC biasing network, vertical bias routing, component reduction, phase-shifter-free beam steering

## Abstract

Dense direct-current (DC) bias routing and numerous radio-frequency (RF) choke inductors pose major challenges to the practical implementation of phase-shifter-free beam steering using PIN-controlled phase switching. To address this issue, a compact vertical DC biasing network is proposed, in which most DC bias lines are routed beneath the ground plane. The DC signals are fed to the PIN diodes through vertical bias lines passing through metallized vias in the dielectric substrate. This arrangement reduces routing congestion and simplifies array-level bias integration. The number of required RF choke inductors is decreased from 112 to 22 per dual-polarized element while preserving the required beam-steering functionality. For experimental validation, a 1 × 3 prototype operating at 3.5 GHz is fabricated and measured. The measured beam directions of −14°, 0°, and +14° agree well with simulations, confirming that the proposed bias network provides the phase control required for beam steering. The proposed network, therefore, offers a compact, low-complexity, and practical solution for scalable phase-shifter-free beam-steering systems.

## 1. Introduction

Beam-steering antenna arrays are widely used in wireless communication, sensing, and base-station systems, where flexible spatial coverage and adaptive radiation control are required [1,2,3,4]. By adjusting the aperture phase distribution, antenna arrays can steer the main beam toward desired directions, improve link quality, and suppress undesired radiation. However, conventional phased arrays usually rely on dedicated phase shifters, multiple RF channels, or complicated feeding networks, which increase insertion loss, hardware cost, calibration effort, and system complexity [5,6]. Therefore, low-complexity beam-steering architectures without conventional phase shifters have attracted increasing attention.

Among these approaches, PIN-diode-controlled phase switching provides a simple way to realize discrete phase states without using conventional phase shifters. In such architectures, direct-current (DC) biasing networks play a critical role by providing the control paths required for phase-state reconfiguration [7]. In practical implementations, dense bias routing and the associated radio-frequency (RF) interference suppression usually necessitate layout complexity and additional lumped components. These challenges make the DC biasing network a key design issue in compact, highly integrated phase-control systems.

Existing phase-control techniques can generally be classified into two categories. One approach relies on multi-port feeding networks, where different ports are selectively excited to synthesize the required phase distribution [8,9,10,11,12]. While effective, such implementations usually rely on multiple RF chains or additional phase-control circuitry, which increases hardware cost and system complexity. Another approach employs PIN-diode-controlled switching networks to achieve beam reconfiguration [13,14,15,16,17]. This approach offers cost-effective control, but its practical implementation strongly depends on the DC biasing network, which is often overlooked in the literature. In compact structures, multiple bias lines are required to drive the switching elements, and unwanted RF leakage along these lines must be suppressed by choke inductors or related isolation components [18]. As a result, the DC biasing network becomes a major source of component overhead and layout complexity in PIN-controlled phase-control systems.

In addition to these phase-control schemes, several simplified and reconfigurable array techniques have recently been investigated to further reduce hardware complexity [19,20,21,22]. Related reconfigurable electromagnetic and switch-based array techniques have also been reported for integrated sensing and communication systems and secure wireless links [23,24]. A phase-modulation approach without phase shifters was reported in [25], in which discrete phase states were controlled by switching PIN diodes through DC biasing lines. However, when RF and DC signals share the same routing path, RF energy can leak into the bias network, dictating numerous RF choke inductors and increasing component count and layout complexity. Therefore, for such PIN-controlled beam-steering architectures, the remaining challenge lies not in the beam-steering principle, but in the realization of a compact, low-complexity DC biasing network.

Here, a vertical DC biasing topology is developed to address the complexity of PIN-controlled beam-steering architectures. Unlike conventional planar bias routing on the radiating layer, the proposed scheme routes most DC bias lines beneath the ground plane, while the PIN diodes (MPP4203-206, Microchip Technology Inc., Chandler, AZ, USA) are biased through vertical DC lines passing through localized metallized vias in the dielectric substrate. This configuration separates most of the bias network from the radiating layer, thereby reducing its direct electromagnetic interaction with the RF radiator (LQG15HS10NG02D, Murata Manufacturing Co., Ltd., Nagaokakyo, Kyoto, Japan) and the need for RF suppression components. Consequently, the number of required RF choke inductors is reduced from 112 (in the previous design [25]) to 22 per dual-polarized element, without compromising the desired beam-steering functionality. In addition, a unified bias-port configuration is adopted to facilitate scalable array-level integration. The feasibility of the proposed topology is validated through a 1 × 3 prototype. The impedance response, radiation patterns, and beam-steering performance are investigated under different switching states over the target operating band of 3.4–3.6 GHz. The measured beam directions of −14°, 0°, and +14° are compared with the simulated results to verify the effectiveness of the proposed biasing topology. Rather than focusing on a new beam-steering principle, this work addresses the practical implementation of a compact and scalable DC biasing network for PIN-controlled phase-shifter-free beam-steering arrays.

## 2. Design of Compact Vertical DC Biasing Network for Phase-Control Structures

### 2.1. Phase-Control Structure

**Figure 1:**
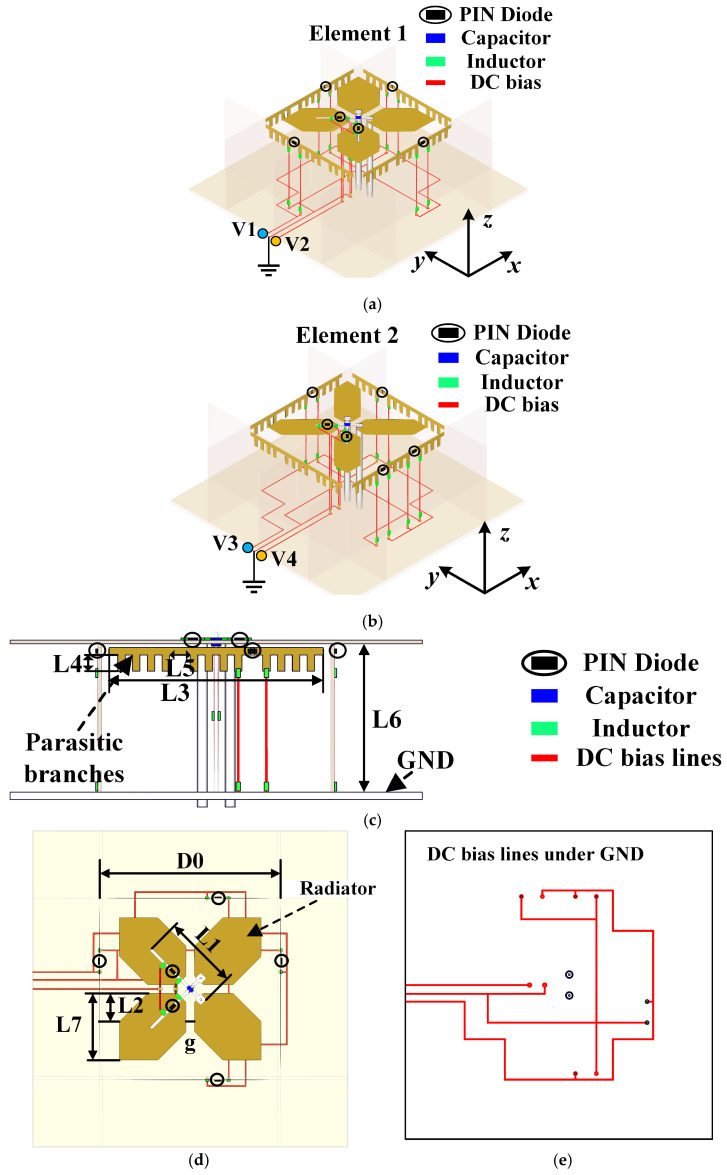
Geometry of the proposed reconfigurable elements. (**a**) Element 1, (**b**) Element 2, (**c**) side view, (**d**) top view, and (**e**) bottom view of the antenna (D0 = 32 mm, L4 = 2.2 mm, L7 = 11.8 mm, L6 = 21.4 mm. Element 1: L1 = 13.2 mm, L2 = 5 mm, L3 = 29 mm, L5 = 2.8 mm, and Element 2: L1 = 10.5 mm, L2 = 7.6 mm, L3 = 27.4 mm, L5 = 0.8 mm).

The proposed phase-control element, shown in Figure 1, integrates PIN-diode-controlled switching branches with the compact vertical DC biasing network. Specifically, Figure 1a and Figure 1b illustrate Element 1 and Element 2, respectively. Each element consists of a radiating element, two PIN-diode-loaded feeding branches, four PIN-diode-loaded parasitic branches, the vertical DC biasing network, and a ground plane. The radiator element and bias network are implemented on an F4B substrate (Taizhou Wangling Insulating Materials Factory, Taizhou, China) (*ε_r_* = 4.2, tanδ = 0.00025, thickness = 0.502 mm). Element 1 and Element 2 share the same basic topology and operating principle, while their geometrical parameters are adjusted to obtain the required phase responses under different switching states. The dimensions of the comb-shaped parasitic branches and the overall antenna height are indicated in Figure 1c. The geometry and detailed dimensions of the radiating element are shown in Figure 1d, while the DC biasing network routed beneath the ground plane is presented in Figure 1e. In the proposed configuration, most DC bias lines are placed below the ground plane and connected to the PIN diodes through localized metallized vias. This arrangement separates the main DC routing network from the radiating layer and reduces the direct interaction between the bias lines and the RF radiator. The key dimensions of the proposed elements are provided in Figure 1, and more details of the original phase-control element can be found in [25]. Full-wave simulations were performed using CST Studio Suite 2020.

The operating states of the element are determined by the switching configurations of the integrated PIN diodes, which enable multiple discrete phase responses. By appropriately controlling the diode states, different phase conditions can be generated without conventional phase shifters, enabling phase synthesis in array applications.

### 2.2. Vertical DC Biasing Network Design

In conventional planar biasing schemes, DC bias lines are typically routed in the same layer as the radiating structure, resulting in insufficient electromagnetic isolation. Consequently, RF currents from the radiator can leak into the bias network and propagate along the DC paths. To suppress this effect, a large number of RF choke inductors are required at the junctions between the bias lines and the radiator, as well as along the return paths, together with DC blocking capacitors (GJM1555C1H5R5WB01D, Murata Manufacturing Co., Ltd., Nagaokakyo, Kyoto, Japan). As a result, the biasing network becomes highly complex. For the considered dual-polarized element, up to 112 RF choke inductors are required for each element [25].

**Figure 2:**
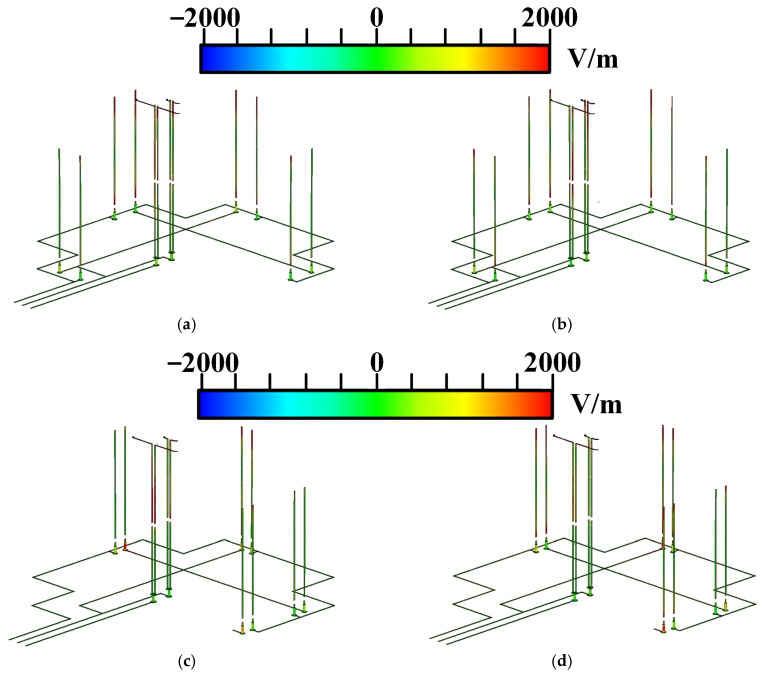
Simulated electric field distributions of the biasing network at 3.5 GHz. Element 1: (**a**) U1D0 and (**b**) U0D1; Element 2: (**c**) U1D0 and (**d**) U0D1.

To reduce this complexity, a vertical DC biasing configuration is adopted, as illustrated in Figure 1 and Figure 2. The DC biasing lines are routed on a dedicated dielectric layer beneath the ground plane, while the PIN diodes are biased through vertical DC paths passing through localized vias, thereby separating the DC network from the radiator. The width of each DC line is set to 0.2 mm to maintain high impedance, and DC blocking capacitors are retained to further suppress residual RF leakage.

Due to the spatial separation between the RF and DC paths, RF leakage into the bias network is significantly reduced, while the ground provides additional electromagnetic shielding. As a result, the number of required RF choke inductors is greatly decreased. In the proposed design, all six PIN diodes share a common ground terminal, while the upper and lower diodes are independently controlled via 2 DC biasing lines. Each element contains three bias ports located on the same side of the substrate, facilitating array-level routing. Consequently, only 22 choke inductors and 2 capacitors are required per dual-polarized element (in contrast to 112 RF choke inductors in the previous design [25]), representing a substantial reduction in circuit complexity.

The simulated electric-field distributions on the DC biasing network under different switching states are shown in Figure 2. The results indicate very weak electric fields along the bias lines, confirming effective electromagnetic isolation between the RF radiator and the DC biasing network.

### 2.3. Implementation and Validation

To verify that the proposed vertical DC biasing network can significantly reduce circuit complexity without compromising antenna performance, this section evaluates both element-level characteristics and array-level beam synthesis. The switching configurations of the integrated PIN diodes determine the phase states of the proposed element. For Element 1, initial phases of 51° and 167° are obtained under the U1D0 (top ON, bottom OFF) and U0D1 (top OFF, bottom ON) states, respectively. For Element 2, the corresponding phase states are 104° and 168°. The selected phase set (51°, 104°, 167°) forms a near-linear phase progression suitable for beam synthesis.

**Figure 3:**
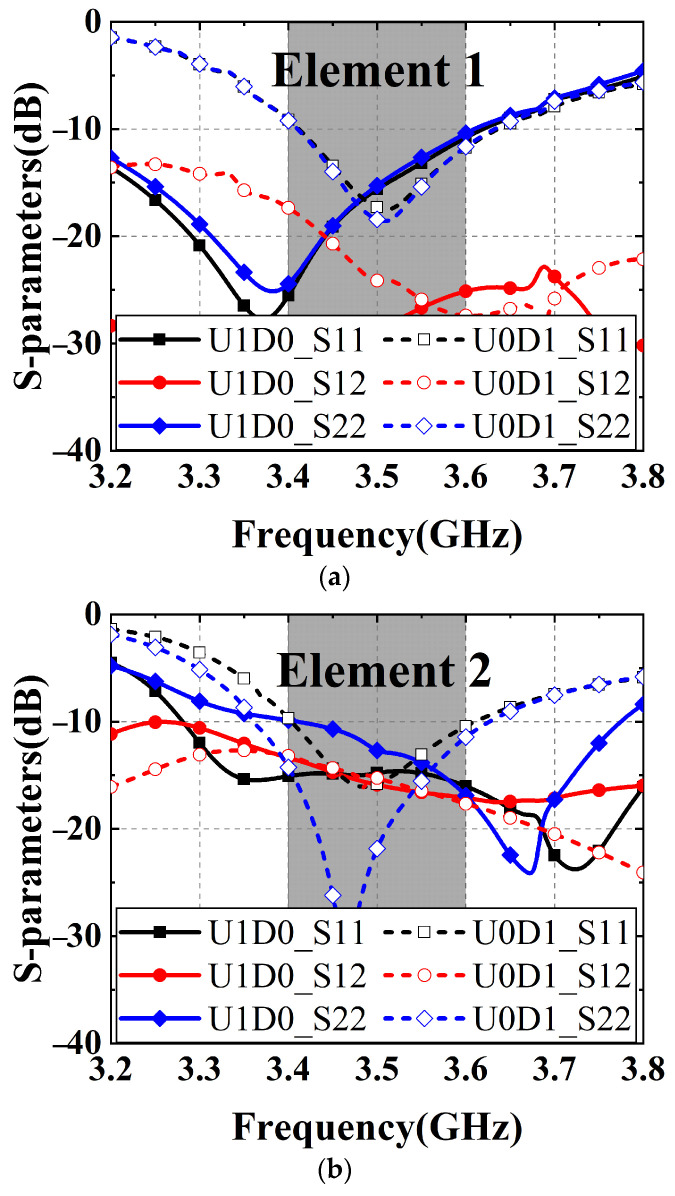

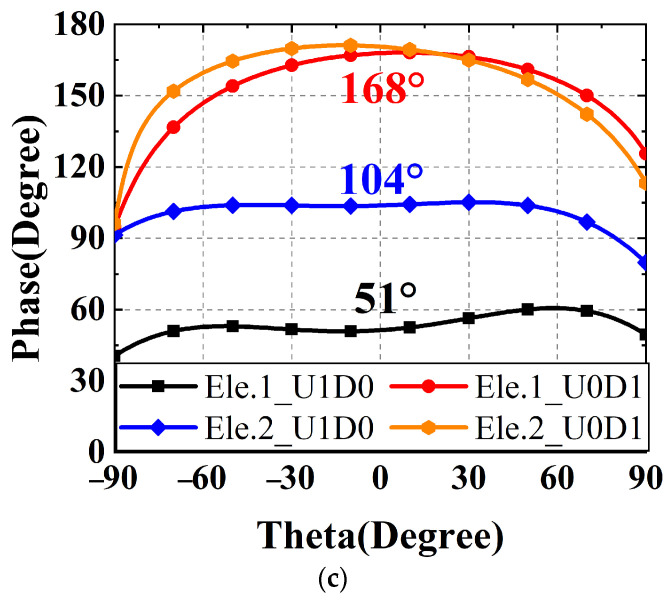
Simulated characteristics of the proposed elements. S-parameters of (**a**) Element 1: U1D0 and U0D1 states; (**b**) Element 2: U1D0 and U0D1 states; and (**c**) phase radiation patterns in the *yoz* plane under different biasing states.

**Figure 4:**
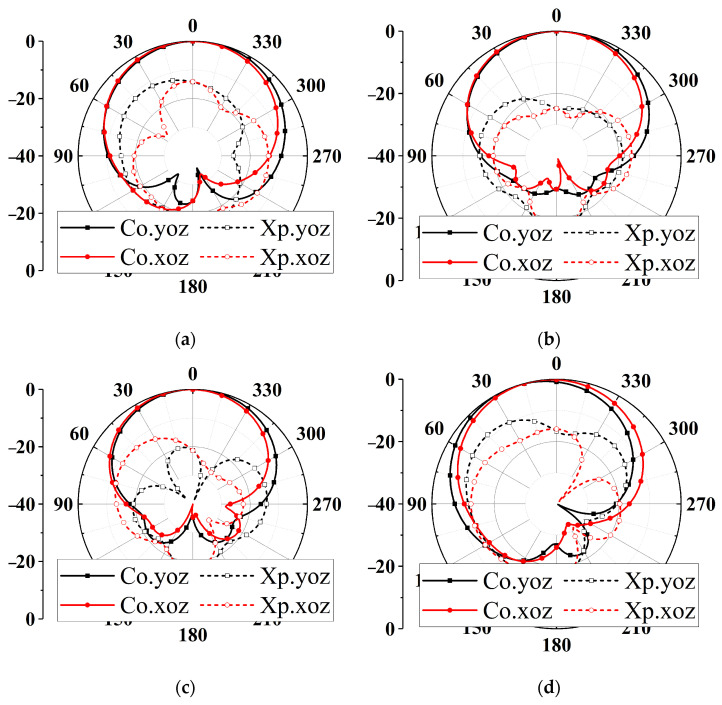
Simulated 2D radiation patterns of the proposed elements at 3.5 GHz. (**a**) U1D0 and (**b**) U0D1 of Element 1; (**c**) U1D0 and (**d**) U0D1 of Element 2.

The simulated S-parameters of the two elements are shown in Figure 3a,b. Good impedance matching is achieved over the 3.4–3.6 GHz band for all switching states. Figure 3c presents the phase distributions in the *yoz* plane under representative biasing states, confirming the availability of discrete phase control. The simulated 2D radiation patterns of the proposed elements at 3.5 GHz are shown in Figure 4. Figure 4a and Figure 4b present the radiation patterns of Element 1 under the U1D0 and U0D1 states, respectively, while Figure 4c,d shows the corresponding results of Element 2. For both elements, the co-polarized radiation patterns remain stable when the switching state is changed, without obvious pattern distortion. The half-power beamwidths in the *y*–*z* plane are 83°, 76°, 83°, and 75°, respectively. In addition, the cross-polarized radiation levels are generally lower than −15 dB for all switching states, satisfying the polarization requirement. These results indicate that the element maintains consistent impedance, radiation, and phase characteristics after introducing the vertical biasing network, making it suitable for array-level beam synthesis.

**Figure 5:**
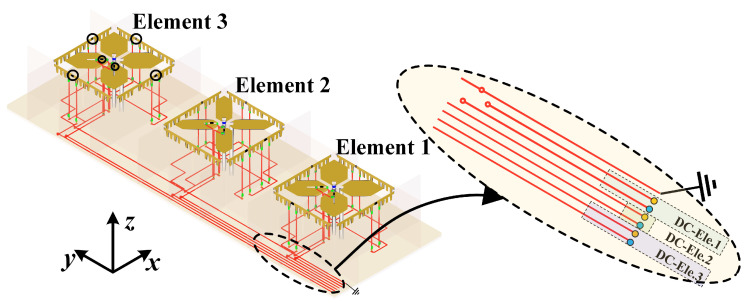
Configuration of the 1 × 3 element phase-control network demonstration. The same color scheme as in Figure 1 is used.

To validate the applicability of the proposed element, a 1 × 3 element configuration is implemented using Element 1, Element 2, and Element 3, as shown in Figure 5. Element 3 has the same physical structure and polarization configuration as Element 1, but is operated under a complementary PIN-diode switching state to form the required phase distribution for beam steering. The inter-element spacing is set to 0.65λ, i.e., a typical value for a vertical base station array [26,27]. All elements share a common ground, while the upper and lower PIN diodes of each element are independently controlled through two DC biasing lines provided by the proposed biasing network.

By switching the DC biasing voltages, the conduction states of the PIN diodes are altered, enabling discrete adjustment of the initial phase for each element. This mechanism enables beam steering by controlling the phase progression across the elements. Three representative phase combinations, including [51°, 104°, 167°], [167°, 168°, 167°], and [167°, 104°, 51°], produce beam directions of −14°, 0°, and +14°, respectively, verifying that the proposed phase-control network can generate controllable phase distributions for beam steering. The beam-steering states are realized by applying different DC-bias states to the PIN diodes. In this work, U1D0 and U0D1 denote the DC-bias states of the PIN diodes loaded on the feeding branches, where “1” and “0” represent the biased and unbiased conditions, respectively. The corresponding DC-bias states for the three beam directions are summarized in Table 1.

**Figure 6:**
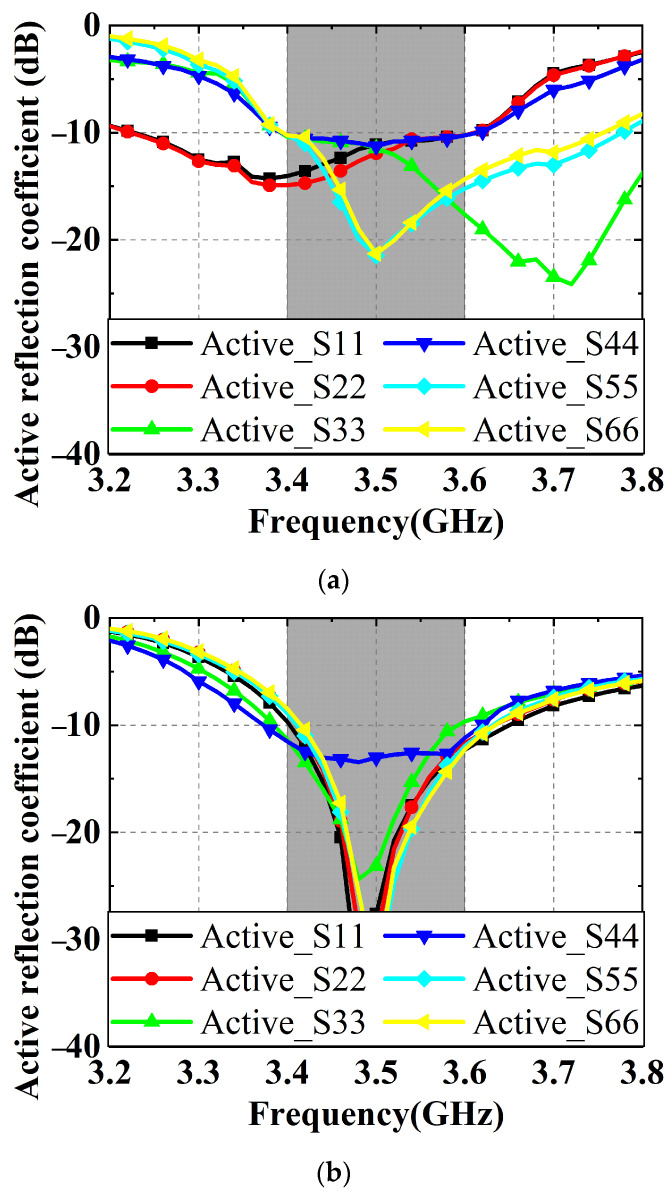
Simulated active reflection coefficients of the 1 × 3 element under two beam states: (**a**) −14° and (**b**) 0°.

To further verify the impedance stability of the 1 × 3 element during beam steering, the active reflection coefficients of all six ports were simulated and are shown in Figure 6. Since Beam State −14° and Beam State +14° are structurally symmetric, only the results of Beam State −14° and Beam State 0° are presented for clarity. As shown in Figure 6, the active reflection coefficients of the six ports remain below −10 dB within the operating band of 3.4–3.6 GHz for both displayed beam states. This indicates that the proposed element maintains stable input matching under different beam-steering states, even when mutual coupling and simultaneous port excitation are considered.

The relative phase differences between adjacent elements determine the beam direction. Constructive interference occurs at specific angles when the required progressive phase difference is satisfied. Therefore, each phase combination corresponds to a predictable beam angle, demonstrating that beam steering can be achieved without conventional phase shifters.

## 3. Fabrication and Measurement Results

**Figure 7:**
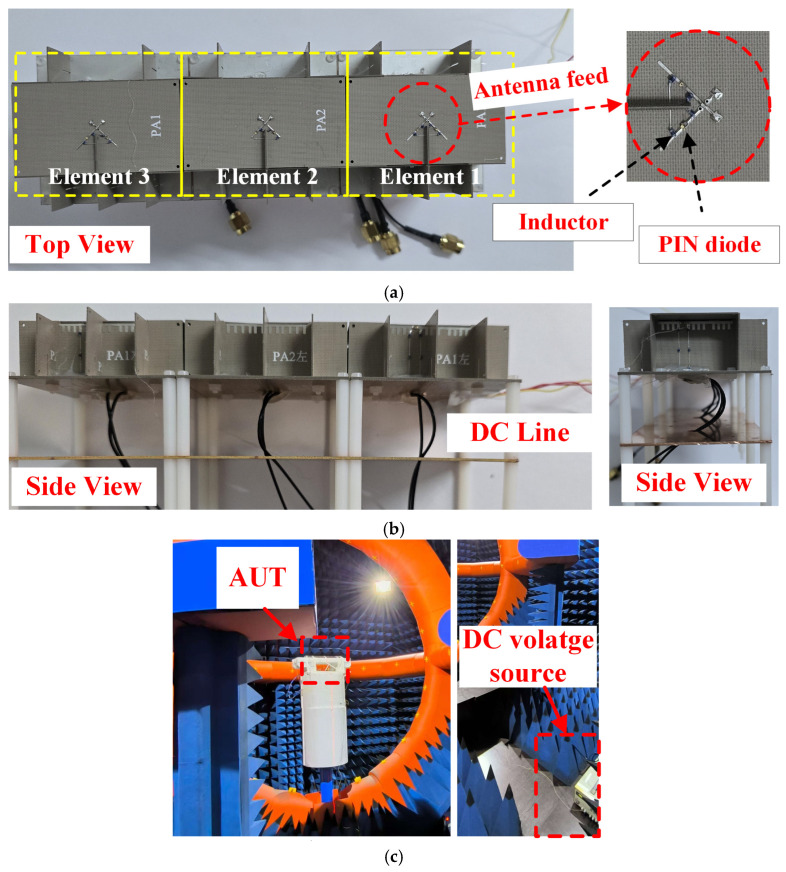
Photographs of the fabricated 1 × 3 element prototype and measurement setup: (**a**) top view, (**b**) side view, and (**c**) anechoic-chamber measurement environment.

To experimentally validate the proposed design, a 1 × 3 element prototype incorporating the vertical DC biasing network was fabricated and tested. The prototype was fabricated by etching the design onto a copper-clad substrate, followed by metallization of the vias. Surface-mount components, including PIN diodes, RF choke inductors, and capacitors, were subsequently soldered onto the substrate. Figure 7 shows the fabricated 1 × 3 element prototype and the measurement setup. Figure 7a presents the top view of the fabricated prototype, where the radiating elements, parasitic branches, PIN diodes, and feeding structures can be observed. Figure 7b shows the side view of the prototype, illustrating the overall profile and the stacked configuration of the antenna. The measurement setup used for the radiation-pattern characterization is also included in Figure 7c.

**Figure 8:**
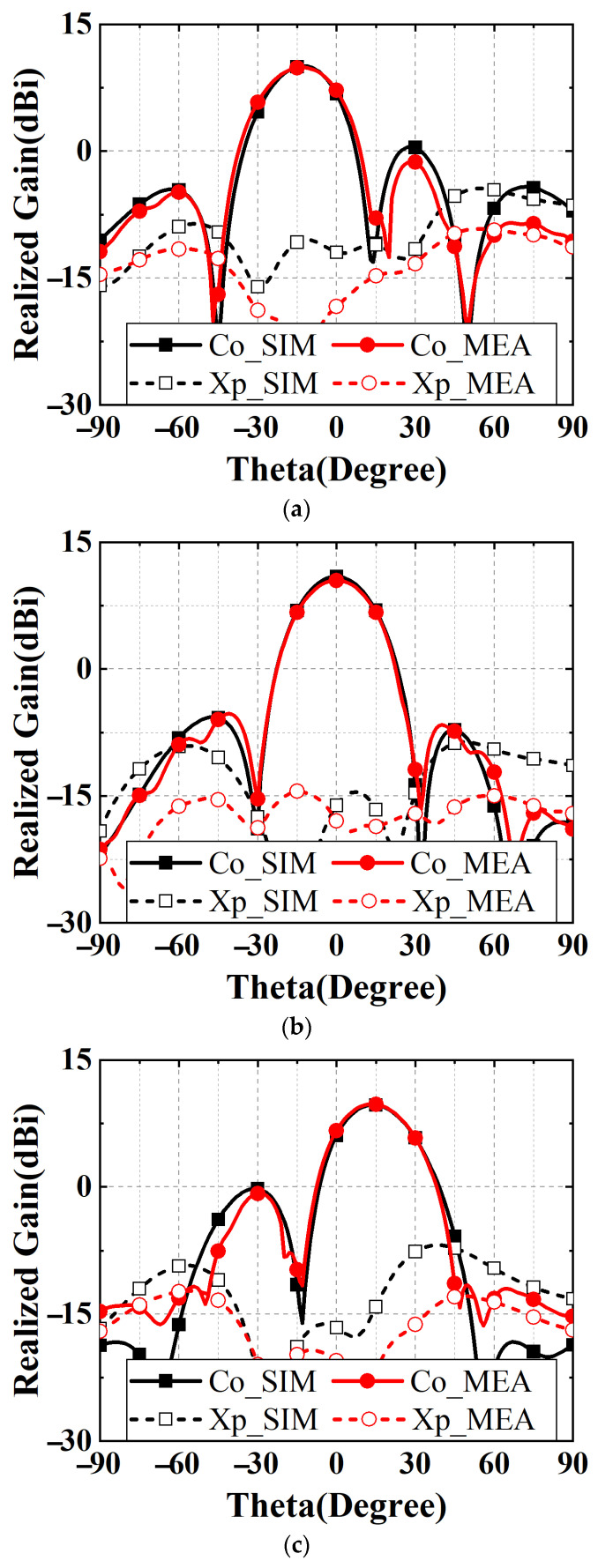
Simulated and measured radiation patterns of the 1 × 3 element at 3.5 GHz under three beam states: (**a**) −14°, (**b**) 0°, and (**c**) +14°.

A 1-to-3 power divider was used to provide equal RF excitation to the three elements. DC biasing voltages were applied via the bias lines to control the switching states of the PIN diodes. Radiation patterns were measured in an anechoic chamber. The measured radiation patterns at 3.5 GHz are shown in Figure 8. By switching the DC bias states, the main beam is steered to −14°, 0°, and +14°, respectively, in agreement with the simulated results. The good agreement between the simulated and measured beam directions indicates that the vertical DC biasing network does not disturb the intended phase progression among the three elements. If strong parasitic coupling or uncontrolled phase shifts were introduced by the bias network, noticeable beam-direction deviations would be expected. The measured pattern shapes also follow the simulated trends, with only minor differences in sidelobe levels, cross-polarization responses, and gain. These discrepancies are mainly attributed to fabrication tolerances, component variations, soldering parasitics of the PIN diodes, RF choke inductors and capacitors, and weak scattering from the measurement cables, DC bias wires, and fixture.

For the three operating states, the simulated gains are 10.08, 10.97, and 9.93 dBi, while the measured gains are 9.90, 10.46, and 9.78 dBi, respectively. The measured total radiation efficiency remains higher than 85% for all beam states. The small gain differences are mainly attributed to the same fabrication and measurement uncertainties discussed above.

## 4. Biasing Network Discussion

The simulated and measured results demonstrate that the proposed vertical DC biasing network preserves the required phase-control and beam-steering functions with fewer RF suppression components. To clarify this improvement from an implementation perspective, Table 2 compares the proposed design with several reported PIN-diode-based phase-control designs.

The comparison includes element size, polarization, number of PIN diodes, number of RF choke inductors, array extensibility, and achievable phase-shift range. The proposed element occupies 0.38λ × 0.38λ and supports dual-polarized operation. With six PIN diodes and 22 RF choke inductors per element, it provides a phase-shift range of 116°. Compared with the reported designs, the proposed biasing topology reduces the number of lumped components while maintaining the required phase-control capability.

Compared with the previous phase-shifter-free implementation in [25], this work keeps the same PIN-controlled beam-steering principle but modifies the DC biasing topology. In [25], many RF choke inductors were needed to suppress RF leakage along the DC biasing paths. In the proposed design, most DC bias lines are routed beneath the ground plane, and the PIN diodes are biased through localized vertical DC paths. Therefore, the number of RF choke inductors is reduced from 112 to 22 for each dual-polarized element, while the required beam-steering states are retained. The main contribution of this work is thus the compact implementation of the DC biasing network, rather than a change in the beam-steering mechanism.

The proposed layout is also convenient for array extension. Since the DC bias lines are routed beneath the ground plane and the bias ports are arranged in a unified manner, routing congestion around the radiating aperture can be reduced. This is particularly useful for multi-element phase-shifter-free beam-steering arrays, where a large number of reconfigurable elements need to be biased and controlled. Therefore, the developed dual-polarized phase-control element is suitable for sub-6 GHz compact base-station arrays requiring discrete beam switching. Its dual-polarized configuration is also compatible with polarization-diversity and MIMO operation, while the electronically reconfigurable radiation behavior may be further used in integrated communication and sensing scenarios requiring adaptive spatial coverage.

From a fabrication viewpoint, reducing the number of lumped biasing components also simplifies assembly. Fewer RF choke inductors lead to fewer soldering points and a simpler DC control network, which is beneficial for large reconfigurable arrays. It should be noted that the present prototype mainly verifies the feasibility of the vertical DC biasing topology and the beam-steering function under small-signal measurement conditions. In future large-scale implementations, bias-line crosstalk, via-density effects, thermal variation of PIN diodes, and manufacturing tolerances should be further considered.

## 5. Conclusions

This paper has presented a compact vertical DC biasing network for a dual-polarized phase-control antenna element. In the proposed design, most DC bias lines have been routed beneath the ground plane, while the PIN diodes have been biased through vertical DC paths passing through localized vias. This configuration has reduced the routing density around the radiating aperture and provided a more regular biasing layout. With this scheme, the number of RF choke inductors required for each dual-polarized element has been reduced from 112 to 22. Meanwhile, the element has maintained good impedance matching, stable radiation patterns, and the required phase-control capability over 3.4–3.6 GHz.

A 1 × 3 element prototype has been fabricated and measured to verify the proposed design. The beam directions have been measured as −14°, 0°, and +14°, showing good agreement with the simulated results. The measured gains have been 9.90, 10.46, and 9.78 dBi for the three beam states, respectively. These results have shown that the proposed vertical DC biasing network has simplified the biasing implementation without noticeably degrading the antenna performance, providing a practical solution for low-complexity phase-shifter-free beam-steering arrays with compact dual-polarized elements and scalable DC control.

## Figures and Tables

**Table 1:** DC-bias states used for the beam-steering cases.

Beam State	Element 1	Element 2	Element 3
−14°	U1D0	U1D0	U0D1
0°	U0D1	U0D1	U0D1
+14°	U0D1	U1D0	U1D0

**Table 2:** Comparison of DC biasing network with reported PIN-based phase-control designs. NA denotes not available.

Ref.	Size (λ^2^)	Pol.	Number of Diodes	Number of Inductors	Array Extension	Phase Shift Range
[14]	0.9 × 0.9	DP	20	Large (240)	No	NA
[15]	0.7 × 0.7	LP	2	Less (24)	Yes	123°
[16]	0.65 × 0.65	LP/CP	16	Middle	No	NA
[17]	0.93 × 0.8	LP	4	Less (26)	Yes	NA
Present	0.38 × 0.38	DP	6	Less (22)	Yes	116°

## Data Availability

All data are available from the author.
